# Wean Earlier and Automatically with New technology (the WEAN study): a protocol of a multicentre, pilot randomized controlled trial

**DOI:** 10.1186/1745-6215-10-81

**Published:** 2009-09-04

**Authors:** Karen EA Burns, Maureen O Meade, Martin R Lessard, Sean P Keenan, Francois Lellouche

**Affiliations:** 1 Interdepartmental Division of Critical Care Medicine, University of Toronto, Toronto, Ontario, Canada; 2 Keenan Research Centre and the Li Ka Shing Knowledge Institute, St. Michael's Hospital, Toronto, Ontario, Canada; 3 Department of Clinical Epidemiology and Biostatistics, McMaster University, Hamilton, Ontario, Canada; 4 Divisions of Anesthesia and Critical Care, Hôpital de l'Enfant-Jésus, Université Laval Quebec City, Quebec, Canada; 5 Division of Critical Care, Royal Columbian Hospital, New Westminster, B.C., Canada; 6 Division of Critical Care, Hopital Laval, Quebec City, Quebec, Canada

## Abstract

**Background:**

Weaning is the process during which mechanical ventilation is withdrawn and the work of breathing is transferred from the ventilator back to the patient. Prolonged weaning is associated with development of ventilator-related complications and longer stays in the Intensive Care Unit (ICU). Computerized or Automated Weaning is a novel weaning strategy that continuously measures and adapts ventilator support (by frequently measuring and averaging three breathing parameters) and automatically conducts Spontaneous Breathing Trials to ascertain whether patients can resume autonomous breathing. Automated Weaning holds promise as a strategy to reduce the time spent on the ventilator, decrease ICU length of stay, and improve clinically important outcomes.

**Methods/Design:**

A pilot weaning randomized controlled trial (RCT) is underway in the ICUs of 8 Canadian hospitals. We will randomize 90 critically ill adults requiring invasive ventilation for at least 24 hours and identified at an early stage of the weaning process to either Automated Weaning (SmartCare™) or Protocolized Weaning. The results of a National Weaning Survey informed the design of the Protocolized Weaning arm. Both weaning protocols are operationalized in Pressure Support mode, include opportunities for Spontaneous Breathing Trials, and share a common sedation protocol, oxygen titration parameters, and extubation and reintubation criteria. The primary outcome of the WEAN study is to evaluate compliance with the proposed weaning and sedation protocols. A key secondary outcome of the pilot RCT is to evaluate clinician acceptance of the weaning and sedation protocols. Prior to initiating the WEAN Study, we conducted a run-in phase, involving two patients per centre (randomizing the first participant to either weaning strategy and assigning the second patient to the alternate strategy) to ensure that participating centres could implement the weaning and sedation protocols and complete the detailed case report forms.

**Discussion:**

Mechanical ventilation studies are difficult to implement; requiring protocols to be operationalized continuously and entailing detailed daily data collection. As the first multicentre weaning RCT in Canada, the WEAN Study seeks to determine the feasibility of conducting a large scale future weaning trial and to establish a collaborative network of ICU clinicians dedicated to advancing the science of weaning.

**Trial Registration Number:**

ISRCTN43760151

## Background

Weaning is the process during which mechanical ventilation is gradually or abruptly withdrawn. In addition, it is the time during which work of breathing is transferred from the ventilator back to the patient until fully autonomous breathing is resumed. Weaning accounts for approximately 40% of the total time spent on mechanical ventilation [1,2]. Invasive mechanical ventilation is associated with the development of important complications including ventilator associated pneumonia (VAP) [3], sinusitis [4], upper airway pathology [3] and respiratory muscle weakness [3]). VAP, in turn, is associated with increased morbidity and a trend toward increased mortality [5]. Mechanical ventilation has recently been identified as a key factor escalating intensive care unit (ICU) costs [6]. For these reasons, minimizing exposure to prolonged ventilation is an important goal of critical care medicine [7].

Over the past decade, research has focused on strategies to limit the duration of ventilation through early identification of weaning candidates [8,10], the conduct of tests of readiness to resume autonomous breathing [spontaneous breathing trials (SBTs)] [11,13] and strategies to reduce support in patients who fail a SBT [14,16]. Several modes and techniques are used to facilitate weaning. The optimal strategy to wean patients from invasive ventilation remains unclear. Compared to traditional care, protocols with their requirement for scheduled surveillance generally decrease the time to discontinuation and total duration of mechanical support [8-10]. Despite demonstration of large-scale implementation, many barriers exist to implementing weaning protocols in clinical practice including the requirement for broad, educational interventions and multidisciplinary compliance with them [17,18].

Automated weaning systems use closed-loop control to interpret clinical data in real time, perform basic and advanced ventilator functions and enable interaction between patients and the ventilator. Closed-loop systems adapt ventilator output by comparing measured values to targeted values of selected respiratory parameters and either minimizing or equilibrating (negative feedback) or amplifying (positive feedback) the differences between these values [19]. SmartCare™ is a unique automated system, specifically designed to guide weaning, that incorporates a closed-loop knowledge based system [20,21] into an automated protocol that adapts the level of pressure support provided to individual patient needs by operationalizing predetermined algorithms based upon respiratory rate (RR), tidal volume (V_T_) and end-tidal carbon dioxide (ETCO_2_).

To initiate SmartCare™ end-users enter the patient's weight, the presence or absence of chronic obstructive pulmonary disease (COPD) or a central neurologic disorder, type of airway prosthesis (tracheostomy or oro/nasal endotracheal tube) and the type of humidification (heated humidification or heat and moisture exchanger) in use. The first three parameters establish limits for RR, V_T _and P_ET_CO_2 _and the latter two items determine the threshold to cycle into a SBT (range: 5 to 12 cm H_2_O). SmartCare™ categorizes patients into one of 8 diagnostic categories based on average measurements of these parameters made every 2 to 5 minutes. With SmartCare™, patients may breathe with a RR ranging from 15 to 30 breaths/min (RR min) (alternatively, 34 breaths/min with neurologic disease) (RR max), a V_T _above a minimum threshold (V_T _Min = 250 ml if weight < 55 kg, or V_T _Min = 300 ml if weight ≥ 55 kg) and a P_ET_CO_2 _below a maximum threshold (max P_ET_CO_2 _= 55 mmHg or max P_ET_CO_2 _= 65 mmHg for COPD patients). SmartCare™ diagnosis a state of normal ventilation when a patient's ventilatory parameters fall within these ranges. If patient parameters fall outside of these ranges, an alternate diagnosis is made and the system adjusts the level of pressure support provided to attain these targets.

SmartCare™ automatically initiates a SBT when predetermined thresholds of pressure support are reached [22,23] in a state of normal ventilation with positive end-expiratory pressure (PEEP) is ≤ 5 cm H_2_O. This period is known as an 'observation period' and varies from 30 minutes to 2 hours in duration. Upon successful completion of a SBT, the ventilator issues a directive stating that the patient is "ready for separation from ventilator". Prior to extubation, physicians must ensure that patients meet readiness criteria to proceed with extubation. With SmartCare™, clinicians titrate the fractional concentration of inspired oxygen (FiO_2_) and PEEP. The automated algorithms may be applied during the day or during the day and at night.

SmartCare™ has been evaluated in physiologic studies and randomized controlled trials (RCTs). Similar to physician assessments using conventional pressure support, SmartCare™ was demonstrated in two physiologic studies to be capable of evaluating patient's ability to breathe spontaneously [22], decrease work of breathing, and reduce periods of respiratory distress during weaning [23]. A prospective cohort study of 42 intubated critically-ill patients, demonstrated that SmartCare™ could support ventilation over prolonged time periods (up to 12 days) [24]. Moreover, the system recognized patient's readiness to undergo a SBT earlier than intensive care physicians [24]. In a preliminary RCT involving 144 patients conducted in 5 European centres, Lellouche and colleagues showed that, compared to usual care directed by protocols in most centres, SmartCare™ decreased the median duration of ventilation from 4 to 2 days (p = 0.02), total duration of ventilation (9 to 6.5 days, p = 0.03), median ICU length of stay (15.5 to 12.0 days, p = 0.02) and nonsignificantly reduced the number of patients requiring prolonged ventilation (>21 days) (15.7 vs 6.7%, p = 0.11) [25]. Conversely, in a single centre RCT in Australia, comparing SmartCare™ to usual care in 102 patients, Rose and coworkers did not demonstrate reductions in weaning time or complication rates [26]. Moreover, the generalizability of results from these regions to Canada where weaning is a collaborative effort, involving registered respiratory therapists (RRTs) and intensivists, is unknown.

### Objectives

The primary objective of the multicentre pilot study comparing SmartCare™ [hereafter referred to as "Automated Weaning"] and "Protocolized Weaning" is to evaluate compliance with the weaning and sedation protocols among intubated patients requiring > 24 hrs of invasive ventilation.

The secondary objectives of the multicentre pilot RCT are to evaluate clinician acceptance of the alternative weaning and sedation protocols using a Visual Analogue Scale daily, and to demonstrate the ability to recruit the desired patient population. In this manner, we will assess the feasibility of conducting a future, definitive weaning RCT in Canada.

## Methods

### Study Design

**Figure 1:**
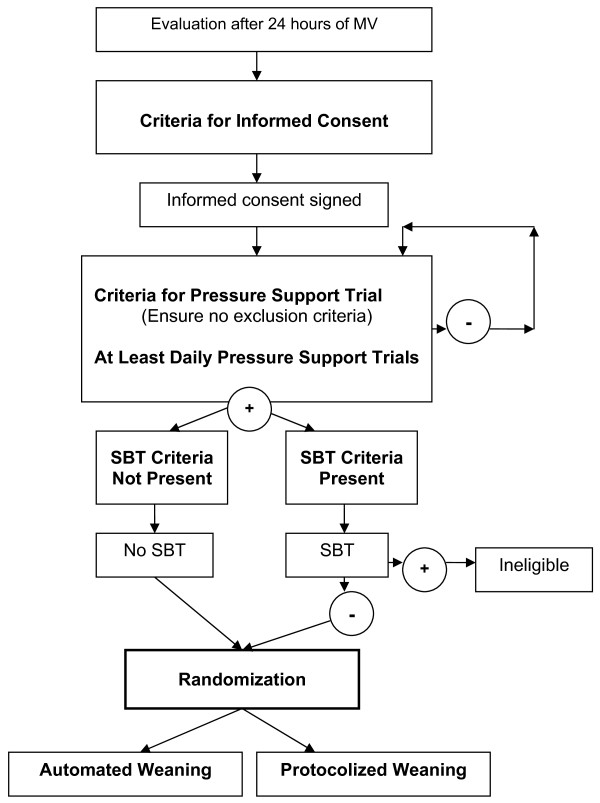
**Study Flow Diagram**.

A pilot RCT is currently underway at 8 adult ICUs across Canada in collaboration with the Canadian Critical Care Trials Group. Ethics approval for the study was obtained from the Research Ethics Board (REB) at St Michael's hospital and the REBs of participating centres. Figure 1 provides an overview of the study.

### Run-in Phase

Prior to initiating the pilot RCT, we conducted a "run-in phase". The run-in phase included two patients at each centre. While, the first patient was randomized to either Automated Weaning or Protocolized Weaning, the second patient was assigned to the alternative weaning strategy in a non-random manner. Upon completion of the two patients, a clinical evaluation committee reviewed the completed case report forms to assess (i) the ability of participating centres to screen and enroll patients, (ii) compliance with study procedures, and (iii) timeliness of data collection and management. Approval from the clinical evaluation committee enabled sites to proceed to enrolling patients in the pilot RCT.

### Patient Screening

A dedicated research coordinator at each site screens patients for eligibility daily between 6:00 and 9:00 am at each site. We use a staged process to (i) identify potential study participants early in the weaning process and (ii) obtain consent for a Pressure Support Trials (PST). If patients fulfill the *Criteria for Consent *and have no exclusion criteria, a member of the investigative team approaches patients or their legal representatives for consent to (i) undergo a PST (once the *Criteria for a Pressure Support Trial *are attained) and a SBT (if criteria are met) and (ii) randomization depending on the outcome of the PST and SBT (see below).

### Criteria for Consent

We developed criteria to enable early identification of potential weaning candidates and to obtain consent early in the weaning process. These criteria include:

1) invasive ventilation for > 24 hours,

2) at least partial reversal of the condition precipitating invasive ventilation,

3) stabilization of "other" organ system failures (i.e. no worsening),

4) pulse oximetry oxygen saturation (SpO_2_) ≥ 90% with fractional concentration inspired oxygen (FiO_2_) ≤ 70%,

5) PEEP ≤ 12 cm H_2_O,

6) weight > 35 kg

7) the absence of exclusion criteria

We exclude patients (i) less than 16 years of age, (ii) with a do not reintubate order documented on chart or anticipated withdrawal of life support, (iii) prolonged cardiac arrest with poor neurological prognosis, (iv) a prior episode of invasive ventilation exceeding 24 hrs during the same hospital stay, (v) tracheostomy, (vi) pregnancy, (vii) known or suspected severe myopathy or neuropathy (i.e., Guillain-Barré syndrome) or quadriplegia, (viii) severe heart failure (grade 3 or 4 left ventricular function or New York Heart Association class 4 dyspnea).

### Criteria for Pressure Support Trial

We consider consented patients, who meet *the Criteria for Consent*, to undergo a PST at least once daily when they meet the following criteria:

1) intubated patient on mechanical ventilation,

2) SpO_2 _≥ 90% with FiO_2 _≤ 50% and PEEP ≤ 10 cm H_2_O,

3) no requirement for high dose vasopressors (i.e., no epinephrine or norepinephrine > 15 µg/min (or 0.2 µg/kg/min) or equivalent dose vasopressin or phenylephrine),

4) motor component of the Glasgow Coma Scale score ≥ 4 (withdraws to pain),

5) stable neurological status (no deterioration in the last 24 hrs, intact respiratory drive, and intracranial pressure < 20),

6) patient not expected to be extubated on the day of study randomization,

7) no surgery or procedure requiring sedation planned in the next 48 hrs.

We reassess criteria for a PST at least once daily after consent is obtained.

### Detailed Study Methods

#### Pressure Support Trials

PSTs are required to identify patients at the onset of the recovery phase when patients are first able to tolerate assisted ventilation. We adjust the level of pressure support provided to obtain a RR ≤ 35 breaths/min during PSTs. The initial level of pressure support is set at 15 cm H_2_O for patients not already on this mode and at the current level of pressure support in patients already on pressure support. PSTs are at least 60 consecutive minutes and not longer than 120 minutes in duration. The maximal pressure support (above PEEP) is 22 cm H_2_O and the minimum is 10 cm H_2_O. A PST can be stopped at any time for sustained hemodynamic or respiratory distress (heart rate ≤ 50 or ≥ 140 beats per minute or new significant dysrhythmias, systolic blood pressure ≤ 80 mm Hg or ≥ 180 mm Hg or RR > 40 breaths/min).

A PST is considered successful if after 60 consecutive minutes without a change in the level of pressure support, the patient remains clinically stable with a RR less than or equal to 35 and greater than 10 breaths/min with no decrease in pulse oximetry saturations (SpO_2 _remains ≥ 90% on an FiO_2 _≤ 50% with PEEP ≤ 8 cm H_2_O). Patients who fail a PST are returned to their previous ventilator settings or settings that restore respiratory comfort. We reassess these patients at least daily for criteria to undergo a PST. Patients who *successfully *complete a PST are assessed for a SBT. Patients who do not meet SBT criteria (i.e., are too early to undergo a SBT) are randomized upon successful completion of a PST.

#### Spontaneous Breathing Trials

Since patients who successfully complete a PST include those who *do *and *do not *require further weaning, we conduct an SBT with the goal of identifying patients who can tolerate pressure support but *fail *an SBT. These individuals are regarded as requiring further weaning. We consider patients with (i) partial or complete reversal of the cause of respiratory failure with SpO_2 _≥ 90% on an FiO_2 _≤ 0.4 (or at baseline level in chronically hypoxemic patients) and PEEP ≤ 5 cm H_2_O, (ii) hemodynamic stability [off vasopressors or on low levels of vasopressors (i.e., levophed ≤ 7 µg/min or ≤ 0.1 µg/kg/min) or equivalent], (iii) absence of uncontrolled sepsis, and (iv) stable haemoglobin > 70 g/L to undergo a SBT. We conduct SBTs of 30 to 120 minutes duration using either a T-piece with oxygen or continuous positive airway pressure (CPAP) ≤ 5 cm H_2_O or PSV of 5 - 7 cm H_2_O (using a heated humidifier or alternatively, 10-12 cm H_2_O with a heat and moisture exchanger) [27,30] with or without 5 cm H_2_O PEEP. Patients who successfully complete a SBT are considered for extubation (i.e., do not require further weaning). Conversely, patients who fail a SBT are randomized. We consider the presence of any one of the following: (i) RR > 35 breaths/min, (ii) clinical signs of respiratory distress (i.e., abdominal paradox), (iii) SpO_2 _< 90% (or below baseline in chronically hypoxemic patients) with FiO_2 _> 50%, (iv) systolic blood pressure ≤ 80 mmHg or ≥ 180 mmHg, (v) heart rate ≤ 50 or ≥ 140 beats/min or new significant dysrhythmias, (vi) severe agitation or diaphoresis, or (vii) Increased somnolence with elevated arterial pressure of carbon dioxide (PaCO_2_) and pH < 7.30 to signify SBT failure. In summary, we randomize consented patients requiring pressure support of at least 10 cm H_2_O who (i) successfully complete a PST, but DO NOT meet criteria to undergo a SBT (i.e., are too early to undergo a SBT) or (ii) who successfully complete a PST and fail a SBT.

#### Study Randomization

Patients undergo stratified randomization within centres based on the presence (or absence) of (i) a diagnosis of known or suspected COPD [31,32] or (ii) a central neurologic disorder, provided that a study ventilator is available. In the event that both diagnoses are present, we prioritize central neurologic disorders during stratification.

#### Weaning Procedures

Randomized patients are initiated on either the Automated Weaning or Protocolized Weaning algorithms until extubation criteria are achieved. All patients will be followed until successful extubation, ICU death, ICU discharge or until 90 days after randomization (deemed ventilator dependent).

##### a) Ventilators

We use the Evita XL ventilator (which includes SmartCare™) or, alternatively, the Evita 2 dura or Evita 4 ventilators with a SmartCare™ ventilator upgrade in patients randomized to the Automated Weaning strategy. We equipped participating centres with a minimum of two of these ventilators. Comparable third generation ventilators (Evita 2 dura, Evita 4, Evita XL, Servo i, Servo 300, Puritan Bennett 840, Puritan Bennett 760, Avea or Galileo) are used in the Protocolized Weaning arm. We preferentially use Evita ventilators in the Protocolized Weaning arm if additional ventilators, when available.

##### b) Humidification systems and ventilator circuits

We clustered use of different forms of humidification to warm inspired air in the WEAN study to gain experience in the interaction of different forms of humidfication with the automated algorithm. We use heated humidifiers (MR850, Fisher & Paykel; Auckland, New Zealand) in both study arms in 5 centres and heat and moisture exchangers at 3 centres, to gain experience with the interaction between humidification and the Automated Weaning system. We record changes from one humidification strategy to another in the event difficulties (secretion volume, alarms etc) on the data collection forms. We use either RT 110 or RT 240 (Evaqua™) circuits in the WEAN study. Reusable circuits are not permitted.

##### c) Ventilator strategies and alarms

Regardless of the strategy to which the patient is randomized, the initial pressure support setting is similar to that used during the PST. In both groups, pressure support may increase or decrease during weaning according to patient needs or events (i.e., mucous plugging, anxiety). We present the details including initiation, titration and discontinuation) of the Automated Weaning and Protocolized Weaning strategies (in Additional file 1, Appendices 1 and 2). We set the maximum inspiratory pressure to 35 cm H_2_O in the Automated Weaning arm.

##### d) Criteria to suspend weaning protocols/return to an alternate mode of ventilation

In both groups, patients are permitted to return to or remain on alternate modes of ventilation for: (i) surgery or invasive procedures requiring sedation, (ii) respiratory distress defined by a) sustained hypoxemia (SpO_2 _< 90%) with FiO_2 _> 60% and PEEP > 10 cm H_2_O or hypercapnia with pH < 7.30 or clinical respiratory distress, b) repeated episodes (≥ 3 episodes within 1 hour wherein an inspiratory pressure (pressure support + PEEP) of 35 cmH_2_O is attained (despite suctioning, bronchodilation etc.), (iii) hemodynamic instability despite fluid boluses and the requirement for high dose vasopressors: norepinephrine > 15µg/min (0.2µg/kg/min) or equivalent, (iv) suspected myocardial ischemia based on electrocardiogram and/or elevated Troponin i, (v) neurologic deterioration with need to control PaCO_2 _(both groups) or alarm indicating "Central hypoventilation" (Automated Weaning), (vi) a requirement for increased sedation resulting in RR < 10 breaths/min. Patients meeting a criterion, are reassessed at least daily with PSTs to identify the earliest time when weaning can be resumed according to the assigned treatment. However, the pre-randomization requirement to undergo a SBT (if criteria are present) is not required at this time point.

##### e) Reintubation and implications for ventilator management

In the Automated Weaning arm, a ventilator capable of delivering SmartCare™ is kept in the patient's room following extubation until the patient is deemed successfully extubated [off non-invasive (NIV) or invasive positive pressure ventilation for 48 consecutive hours]. Patients remain on the assigned strategy until ICU discharge, ICU death, successful extubation or until day 90 following randomization. Patients requiring reintubation in the Automated Weaning arm within 48 hours of extubation will be weaned using SmartCare™ as soon as possible (i.e., upon successful completion of a PST). All patients that require reintubation after successful extubation will be ventilated according to usual practice.

##### f) Sedation titration

We developed a sedation guide to guide sedation administration and limit performance bias during weaning. Critical care nurses titrate sedation to achieve either a Sedation Agitation Scale (SAS) [33] score of 3 to 4 (see Additional file 1, Appendix 3) or a Richmond Agitation Scale Score (RASS) [34] of -3 to 0 [35]. Sedation titration is not mandatory in patients meeting criteria to return to or remain on an alternate mode of ventilation.

##### g) Other considerations

We developed a PEEP/FiO_2 _chart to guide their titration during weaning (see Additional file 1, Appendix 4). Use of the PEEP/FiO_2 _chart is not mandatory for patients meeting criteria to return to or remain on an alternate mode of ventilation.

Additional important considerations in developing the WEAN Study protocol include (i) the initiation and use of NIV following extubation [36,37], (ii) reintubation and reinitiation of weaning [38] and (iii) performance of a tracheostomy (Additional file 1, Appendix 5). For patients randomized to Automated Weaning, the data entered to initiate the system *mus*t be re-entered following a tracheostomy or change in humidification device prior to reconnection.

#### Study Outcomes

The primary outcome of the WEAN pilot study is compliance with the weaning and sedation protocols. Secondary outcomes include (i) daytime clinician (nurse; RRT and physician) acceptance of the sedation and weaning protocols, respectively, (ii) time to successful extubation defined as the time from randomization to unsupported (no requirement for NIV or invasive ventilation), spontaneous breathing for ≥ 48 hours after extubation (or disconnection with a tracheostomy), (iii) time to completion of a successful SBT, (iv) total duration of mechanical ventilation (time from intubation to termination of ventilation), (vi) ICU and hospital length of stay, (vii) ICU and hospital mortality, and the proportion of patients (viii) developing nosocomial pneumonia [39], (ix) with weaning (self-extubation, tracheostomy, reintubation and prolonged mechanical ventilation >21 days) or ICU (myocardial infarction and pneumothoraces) complications, and (x) requiring NIV. Any NIV use is considered clinically relevant.

#### Data Collection

We collect data at ICU admission, study inclusion and daily thereafter. Whereas protocol violations (non compliance) in the Automated Weaning arm include return to/remain on an alternate mode of mechanical ventilation in the absence of meeting criteria and any unauthorized interruptions (cessations, manual increases/decreases) in the automated protocol, violations in the Protocolized Weaning group include unauthorized use of modes other than pressure support (i.e., assist control) without criteria to return to or remain on an alternate mode of mechanical ventilation. We tabulate a daily score reflecting the number of SBTs conducted divided by the number of required SBT assessments in which all criteria were met in the Protocolized Weaning group. Sedation protocol compliance is assessed by the percentage of daily SAS scores < 2 or > 5 (or alternatively, RASS scores < - 4 or > +1) in both arms. We also evaluate compliance with the PEEP/FiO_2 _chart in both groups. RRT and physician acceptance of the weaning protocols and critical care nurse acceptance of the sedation protocol is assessed daily using Likert [40] scales [ranging from 0 highly unacceptable (extremely difficult to use) to 10 highly acceptable (extremely easy to use)].

#### Statistical Analyses

Descriptive statistics including means, standard deviations, medians, interquartile ranges and frequency distributions will be used to summarize the data. For univariate analyses, we will use the Chi-square test (alternatively, Fisher's exact test when the expected value is ≤ 5) and Student's t-test (alternatively, the Mann-Whitney U-test, if normality assumptions are not satisfied) for binary and continuous outcomes, respectively. We will evaluate the percentage of (i) hours off-protocol and (ii) daily SAS scores < 2 or > 5 (or RASS equivalent) by treatment group. Compliance will be considered acceptable if ≥ 75% [that is; protocol violations (off protocol hours without meeting criteria to return to/remain on an alternate mode of mechanical ventilation) or SAS scores < 2 or > 5 (equivalent RASS -4 < or > +1) occur < 25% of the time]. SBT compliance will be considered acceptable if ≥ 75% of SBT opportunities are realized. We will compare average Likert scale scores based on clinician group and the weaning strategy utilized using Analysis of Variance (ANOVA). We will compare time to successful extubation and death between groups using time-to-event analysis with censoring of deaths and application of the log-rank test. We will consider p-values ≤ 0.05 to be statistically significant.

#### Sample Size

Estimates are not available to allow precise sample size estimation of the primary outcome for the multicentre pilot RCT. To ensure that the study protocols are easy to follow before engaging in a definitive weaning RCT, we estimate that a total of 90 patients (ideally approximately 11-12 patients per site) are required to assess protocol compliance. To ensure that all centres gain some experience with both protocols, we set a maximum site enrolment of 35 patients.	

## Discussion

Life support technology interventions account for 5-10% of acute care bed occupancy and 34% of hospital budgets [41]. Current demands are expected to escalate in the future as the middle sector of our population ages [42]. Since up to 40% of the time on mechanical ventilation is related to weaning, Automated Weaning holds promise as a strategy to reduce ICU length of stay and the total time spent on mechanical ventilation by reducing weaning time, and weaning and ICU-related complications. More importantly, Automated Weaning may reduce the burden of illness related to protracted invasive ventilation and improve patient-important outcomes [43]. A key benefit of SmartCare™ is that weaning is unencumbered by limited clinician availability in the busy ICU setting. Prior to conducting a full scale RCT comparing the effect of the alternative weaning strategies on important clinical outcomes, we designed a feasibility RCT to assess clinician compliance with and acceptance of the detailed weaning and sedation protocols [44].

In the absence of evidence regarding the current 'standard of care in weaning' in Canada, we developed, tested and administered a questionnaire to RRT and physician leaders using rigorous survey methodology [45] to identify key aspects of weaning (use of protocols, daily screening, conduct of SBTs, preferred methods of support) at Canadian teaching hospitals. In this manner, we identified that daily screening and pressure support were common features of weaning and that most centres conduct SBTs using one of three strategies [46]. The results of the National Weaning Survey informed the design of the paper-based weaning protocol (control arm) in the WEAN Study.

Prior to initiating the multicentre WEAN Study, we conducted a run-in phase study involving two patients per centre. We randomized the first participant at each centre to either Automated or Protocolized Weaning and assigned the second patient to the alternate weaning strategy. We conducted the run in phase to ensure that participating centres could implement the detailed weaning and sedation protocols and complete the case report forms. Mechanical ventilation studies require detailed daily data collection and for protocols to be operationalized continuously. Following review of run in phase study procedures, a clinical evaluation committee approved sites to proceed with enrollment in the WEAN pilot RCT.

## Conclusion

The WEAN pilot study was designed to evaluate clinician compliance with and acceptance of the proposed weaning and sedation protocols. In addition, we will ascertain whether centres can recruit the desired patient population. Strengths of the WEAN Study include the use of central randomization, allocation concealment, stratification (to ensure equal distribution of conditions that may prolong ventilation) and the use of protocols in both arms to limit treatment and performance bias. To this end, both treatment arms use pressure support and include opportunities to undergo SBTs. Moreover, they share a common PEEP/FiO_2 _chart, sedation protocol, extubation and reintubation criteria, and criteria for NIV use for post-extubation respiratory failure. The major threat to the validity of the pilot RCT is that it is, out of necessity, unblinded. To this end, we developed detailed weaning and sedation protocols to limit potential sources of bias and standardize weaning and sedation administration in both study arms and across participating centres. While we can not blind clinicians, patients and data collectors, we will blind the data analyst to treatment group assignment until the planned statistical analyses are completed. As the first multicentre weaning RCT in Canada, the WEAN Study seeks not only to determine the feasibility of conducting a large scale future weaning trial, but to establish a collaborative network of ICU clinicians dedicated to advancing the science of weaning.

## Abbreviations

**VAP**: ventilator associated pneumonia; **ICU**: Intensive Care Unit; **SBT**: Spontaneous Breathing Trial; **RR**: Respiratory rate; **V_T_**: Tidal volume; **ETCO_2_: **end tidal carbon dioxide; **COPD**: Chronic Obstructive Pulmonary Disease; **PEEP**: Positive End-Expiratory Pressure; **FiO_2_**: Fractional concentration of inspired oxygen; **RCT**: randomized controlled trial; **SpO_2_**: Pulse oximetry oxygen saturation; **CPAP**: continuous positive airway pressure; **PST**: Pressure Support Trial; **NIV**: noninvasive ventilation; **SAS**: sedation agitation scale; **RASS**: Richmond Agitation Scale Score; **PaO_2_/FiO_2_**: Arterial partial pressure oxygen/Fractional concentration of inspired oxygen ratio; **RRT: **Registered Respiratory Therapist; **ASB**: automatic spontaneous breathing; **HME**: heat and moisture exchanger; **HH**: heated humidification; **ATC**: automatic tube compensation; **LOC**: level of consciousness.

## Competing interests

Drs Burns and Lellouche hold a travel bursary from Draeger Medical Inc. (Canada) to conduct site visits for the WEAN Study. The WEAN Study is an investigator-initiated, peer-review funded trial. No other author(s) has a financial relationship with a commercial entity that has an interest in the subject of the manuscript.

## Authors' contributions

KB, MM, ML, SK, and FL contributed to the conception and design of the WEAN study, drafted and revised the manuscript for important intellectual content.

KB, MM, ML, SK, and FL approved the final version of the manuscript.

## Supplementary Material

Additional file 1 Appendices 1-5. Click here for file
