# Supplementary material for: Wean Earlier and Automatically with New technology (the WEAN study): a protocol of a multicentre, pilot randomized controlled trial
Source: Trials. 2009 Sep 4;10:81. doi: 10.1186/1745-6215-10-81 (PMC2749823; doi:10.1186/1745-6215-10-81)
Supplement: Additional file 1 — Appendices 1-5. [file 1745-6215-10-81-S1.doc]

**Appendix 1: Automated Weaning Protocol**

Initiation:

STEPS FOR EVITA XL SET UP:

Applies to Automated Weaning patients following: (1) randomization, (2) reinitiation after interruption, (3) reintubation *within* 48 hrs (4) reinitiation after tracheostomy.

Step 1: Switch to “Pressure Support” (CPAP/ASB)

Step 2: Select “SmartCare” tab

Step 3: Enter patient weight (measured body weight)

Step 4: Type of humidification at randomization: HH or HME based on clusters.

Step 5: Airway access: endotracheal tube (oro or nasal) at randomization

or tracheostomy (after randomization)

Step 6: Known or suspected COPD – yes/no

Neurologic disorder (only central neurologic disorder) – yes/no

Step 7: Night rest – select “no”

Step 8: Set alarms:

Maximum Inspiratory Pressure: 35 cm H_2_O

Max RR 40 b/min

ETCO_2_ limits: 15 mm Hg (minimum) and 70 mm Hg (maximum)

Step 9: You must ensure the following prior to initiating a session

a) ETCO_2_ monitoring is “activated”

b) Check that the ETCO_2_ cuvette (connector) is clean on the interior aspect

c) Flow Monitoring is “activated” and

d) ETCO_2_ is calibrated

Step 10: Apnea ventilation must be “activated” and set

Step 11: ATC (Automatic Tube Compensation) must be “deactivated”

Step 12: “Leak compensation” must be activated

Before initiating mechanical ventilation with SmartCare™, baseline information including [patient measured body weight, presence or absence of COPD/central neurologic disorder, type of airway prosthesis (tracheostomy or endotracheal tube) and humidification used [heat and moisture exchanger (HME) or heated humidification (HH)] is entered using the touch pad ventilator interface. These items are required to determine limits for V_T_, PETCO_2_ and the minimal level of pressure support to initiate an SBT (range: 5 - 12 cm H_2_O; with HH 5 - 7 cm H_2_O and HME 9-12 cm H_2_O). Pressure support, PEEP and FiO_2_ are initiated as per the levels used in the PST.

Titration: Pressure support be continuously adapted (by averaging RR, V_T_, PETCO_2_ every 2 to 5 minutes) with RRs [ranging from 15 to 34 breaths/min, minimum V_T_ of at least 300 cc (250 cc if patient’s weight < 55kg)] and maximum P_ET_CO_2_ of 55 mm Hg (or 65 mm Hg for COPD patients). When ventilatory parameters fall within these constraints, a normal ventilatory state is diagnosed. Conversely, the system adjusts the level of pressure support to attain these targets when ventilatory parameters fall outside of these constraints. Specifically, the automated system increases pressure support by 2 cm H_2_O in response to tachypnea (RR> 30 breaths/min or 34 breaths/min with a central neurologic disease) and by 4 cm H_2_O (RR > 36 breaths/min) and lowers the pressure support delivered by 2 cm H_2_O when bradypnea (RR< 12 breaths/min) is diagnosed with no increase in PETCO_2_. When V_T_ or PETCO_2_ are out of the defined ranges, insufficient ventilation is diagnosed and pressure support is increased by 2 cm H_2_O. Patients are weaned at night and during the day. If ventilation becomes unstable the mechanical assistance provided will be augmented. Specific management strategies depend on the duration of instability and the level of pressure support required previously. PEEP and FiO_2_ are titrated to maintain PaO_2_ 60- 80 mm Hg (or at baseline levels in hypercarbic patients) or SpO_2_ 90 – 95%%. A PEEP/FiO_2_ chart is used to titrate FiO_2_ and PEEP during weaning (see Appendix 4). We assess and document ventilator parameters at least every 4 - 6 hrs. Sedation is titrated to either a SAS score of 3-4 or a RASS score of -3 to 0 during weaning.

Discontinuation: SBTs are automatically conducted when a minimum level of pressure support is reached [5 cm H_2_O (HH) with a tracheostomy or 7 cm H_2_O (HH) with an oro/nasotracheal airway] and PEEP has been reduced to 5 cm H_2_O. Alternatively, SBTs are automatically conducted when a minimum level of pressure support is reached [9 cm H_2_O (HME) with a tracheostomy or 12 cm H_2_O (HME) with an oro/nasotracheal airway] and PEEP has been reduced to 5 cm H_2_O. SBT duration (60 or 120 minutes) is contingent upon the level of pressure support initially used and the magnitude of the decrease in pressure support (ranging from 2 to 4 cm H_2_O) tolerated. If the initial pressure support required was <20 cm H_2_O, the SBT duration is 60 minutes and if the initial pressure support required was > 20 cm H_2_O, the SBT duration is 120 minutes. The level of pressure support used during the SBT is the “minimal level of pressure support” delivered during automated ventilation. This level will remain steady as long as the patients breathing pattern remains stable. If more assistance is required, the SBT will be terminated and pressure support will be increased. Alternatively, if less support is required (ventilatory diagnosis of hyperventilation) pressure support will decrease to 5 cm H_2_O. When a successful SBT is completed, a directive is issued by the ventilator informing staff to “consider separation from the ventilator”. In the event of a successful SBT in patients with an endotracheal tube patients must be assessed for extubation. If the message appears and extubation criteria are fulfilled then patients must be extubated immediately (must be performed within 15 hours of meeting the extubation criteria). After an unsuccessful SBT, ventilator settings are adjusted to restore respiratory comfort until criteria are met to undergo a subsequent SBT.

Extubation Criteria: Patients are assessed for extubation criteria after successful completion of a SBT. To be extubated patients must have:

1. An SpO_2_ > 90% on an FiO_2_ < 40% and PEEP< 5 cm H_2_O (or at baseline level in chronically hypoxemic patients)

2. A cough of sufficient strength to clear secretions and must not require suctioning more than every 2 hours.

3. No procedures requiring sedation or surgery should be planned within 48 hours after extubation

4. Patients should be hemodynamically stable (off vasopressors or on minimal levophed i.e.

< 7 ug/min or equivalent) and

5. A level of consciousness sufficient to ensure airway protection.

Disconnection criteria (tracheostomized patients): All of the above criteria, except #5 above, apply to patients with a tracheostomy who successfully complete a SBT. Disconnection from the ventilator must be performed immediately after meeting criteria # 1 - 4 above.

Peri-Extubation Ventilator Management:

*If a patient **passes an SBT** and **meets Extubation Criteria**: (s)he should be immediately extubated (and must be extubated within 15 hrs).

*If a patient **passes an SBT** and **does not meet Extubation Criteria**: (s)he should continue on pressure support 5 - 7 cm H_2_O ± PEEP < 5 cm H_2_O (HH) or 9-12 cm H_2_O ± PEEP < 5 cm H_2_O (HME). Patients should be continued on SmartCare pressure support. Pressure support will be titrated automatically.

*If a patient **fails an SBT** – the assigned weaning protocol should be resumed.

Ventilator Management After Extubation: Protocols continue until successful extubation (at least 48 hrs of unassisted spontaneous breathing), ICU death, ICU discharge or until day 90 after randomization.

The AW ventilator must remain in the patient’s room until successful extubation or ICU discharge (which ever comes first).

**Appendix 2: Protocolized Weaning**

Initiation: Pressure support is the recommended mode of ventilation. Pressure support, FiO_2_ and PEEP are initiated as per the levels used in the Pressure Support Trial.

Titration: The level of pressure support is reevaluated at least every 4 - 6 hrs and titrated to avoid respiratory distress (use of accessory muscles or RR > 30 breaths/min (alternatively, > 34 breaths/min in patients with COPD or a central neurologic disease) or overassistance. Patients are weaned during the day and at night. PEEP and FiO_2_ are titrated to maintain PaO_2_ 60- 80 mm Hg (or at baseline levels in hypercarbic patients) or SpO_2_ 90 – 95%%. A PEEP/FiO_2_ chart is used to titrate FiO_2_ and PEEP during weaning. We assess and document ventilator parameters at least every 4 – 6 hrs. Sedation is titrated to either a SAS score of 3-4 or a RASS score of -3 to 0 during weaning.

Discontinuation: SBTs are performed when the following criteria are met on pressure support:

a) The cause of respiratory failure is partially or completely reversed with SpO_2_ ≥ 90% on an FiO_2_ ≤ 0.4 (or at baseline in chronically hypoxemic patients) and PEEP ≤ 5 cm H_2_O,

b) The patient is hemodynamic stable [off vasopressors or on low levels of vasopressors (i.e. levophed ≤ 7µg/min or ≤ 0.1µg/kg/min) or equivalent],

c) Absence of uncontrolled sepsis,

d) Hemoglobin > 70 g/L and stable,

The above criteria are evaluated at least once per day. SBTs are performed using either a T-piece (or Trach Mask) with oxygen or CPAP (< 5 cm H_2_O) or pressure support 5 - 7 cm H_2_O ± PEEP < 5 cm H_2_O (HH group) or 10 - 12 cm H_2_O ± PEEP < 5 cm H_2_O (HME group). All SBTs are 30 - 120 minutes in duration.

SBT failure/Termination criteria is defined by the presence of any ONE of

(i) RR > 35 breaths/min,

(ii) Clinical signs of respiratory distress (i.e. abdominal paradox),

(iii) SpO_2_ < 90% (or below baseline in chronically hypoxemic patients) with FiO_2_ > 50%,

(iv) Systolic blood pressure ≤ 80 mmHg or ≥ 180 mmHg,

(v) Heart rate ≤ 50 bpm or ≥ 140 beats/min or new significant dysrhythmias,

(vi) Severe agitation or diaphoresis,

(vii) Increased somnolence with elevated PaCO_2_ and pH < 7.30.

After an unsuccessful SBT, ventilator settings are adjusted to restore respiratory comfort until criteria are met to undergo a subsequent SBT. For patients who fail an SBT, at least one SBT is attempted daily. In the event of a successful SBT in patients with an endotracheal tube, patients must be must be extubated immediately (must be performed within 15 hrs of meeting the extubation criteria). Alternatively, in the event of a successful SBT in a tracheostomized patient the patient must be disconnected.

Extubation Criteria: Patients are assessed for extubation criteria after successful completion of an SBT. To be extubated patients must have:

1. An SpO_2_ > 90% on an FiO_2_ < 40% and PEEP < 5 cm H_2_O (or at baseline level in chronically hypoxemic patients)

2. A cough sufficiently strong to clear secretions and not requiring suctioning more than every 2 hours.

3. No procedures requiring sedation or surgery planned within 48 hours after extubation

4. Patients should be hemodynamically stable [off vasopressors or on minimal levophed i.e.

< 7 ug/min (or 0.1 ug/kg/min) or equivalent] and

5. A level of consciousness sufficient to ensure airway protection (not required for tracheostomized patients).

Disconnection criteria (tracheostomized patients): All of the above criteria, except #5 above, apply to patients with a tracheostomy who successfully complete a SBT. Disconnection from the ventilator must be performed immediately after meeting criteria # 1 - 4 above.

Peri-Extubation Ventilator Management:

*If a patient **passes an SBT** and **meets Extubation Criteria**: (s)he should be immediately extubated (and must be extubated within 15 hrs).

*If a patient **passes an SBT** and **does not meet Extubation Criteria**: (s)he should continue on pressure support 5 - 7 cm H_2_O ± PEEP < 5 cm H_2_O (HH) or 10-12 cm H_2_O ± PEEP < 5 cm H_2_O (HME). Patients should be continued on these settings. Pressure support and PEEP should be titrated to avoid respiratory distress or over assistance at least Q4-6 hr.

*If a patient **fails an SBT** – the assigned weaning protocol should be resumed.

Ventilator Management After Extubation: Protocols continue until successful extubation (at least 48 hrs of unassisted spontaneous breathing), ICU death, ICU discharge or until day 90 after randomization.

**Appendix 3: SAS Sedation Protocol**

**(** A RASS sedation protocol was also developed using comparable sedation scores)**

Sedation and/or analgesia are available to all participants in whom it is required. We administer sedation and analgesia using a nurse-implemented sedation protocol. The goals of sedation administration for all study participants are to (i) minimize agitation, (ii) promote ventilator synchrony, (iii) relieve anxiety and pain and (iv) avoid inappropriate oversedation. Critical Care nurses titrate sedatives to achieve a calm, cooperative patient [Sedation Agitation Scale of 3 (acceptable) or 4 (preferred)] [34,35] who is capable of following simple commands (i.e. hand squeezing to command). We do not assess strict adherence to the protocol, but rather, whether the goals of the protocol are achieved. Nurses evaluate SAS scores at least every 4 hrs or at least every 2 hours if dose changes were made on the prior measurement. We record SAS scores on the case report forms every 4 hours. Sedation titration is not mandatory if the patient is on an alternate mode of MV Administration of sedation and analgesia during weaning is according to the following principles:

**General Principles:**

1. The goal is to attain a SAS scale score of 3 or 4; that is, to avoid oversedation or undersedation.
2. Attempts are made to distinguish the etiology of agitation during weaning and to administer appropriate treatment with either sedatives (anxiolytics) or analgesics or both.
3. Regardless of the medication(s) administered, the amount administered (per dose and total daily dose) is individualized and is expected to vary from patient to patient and within patients over time.
4. If possible, attempts are made to minimize sedation/analgesia over time and to minimize the use of continuous IV sedation/analgesia.

**Oversedation (SAS scores of 1 or 2)**

1. In the presence of either intermittent or continuous sedation/analgesia and a SAS score of 1 or 2, sedation/analgesia **except** in the presence of ventilator dyssynchrony or signs of respiratory distress refractory to supportive measures (e.g., suctioning, bronchodilation, diuresis).
2. If a SAS score > 3 is obtained within 8 hrs after the sedation/analgesia was stopped, a physician should judge whether the patient requires ongoing sedation/analgesia.
3. If the patient is judged to require additional sedation/analgesia, intermittent or continuous sedation/analgesia should be resumed at half the previous dose and adjusted accordingly thereafter to attain a SAS score of 3 to 4 at the discretion of the attending physician.

**Optimal Sedation (SAS scores of 3 to 4)**

1. Once SAS scores of 3 to 4 have been obtained, a judgment should be made as to whether the patient requires ongoing sedation/analgesia.
2. If ongoing sedation/analgesia is required, routes of administration, doses administered and dosing intervals can be maintained and preferably decreased. The goal should be to minimize the amount and duration of sedation/analgesia administered over time.

**Undersedation (SAS scores of 5, 6 and 7) without continuous IV sedation**

1. Preferential and sparing use of small doses of intravenous short-acting sedatives (i.e. Midazolam; range: 1 to 5 mg) and analgesics (i.e., Fentanyl; range: 25 to 75 μg).
2. When short-acting agents are required more than every 4 hrs, small doses of longer-acting sedatives, (i.e., Lorazepam 1 to 4 mg or or Clonazapem 0.5 to 2.0 mg q 8h) and analgesics (i.e. Morphine 2 to 4 mg q 4-6h), administered IV or orally is encouraged.
3. Infusions of sedative or analgesic agents are reserved for patients refractory to long acting agents or requiring frequent boluses (> every 2 hours) of short-acting agents or persistently attaining SAS scores > 5.
4. When required, continuous infusions of sedative/analgesic agents are titrated to achieve SAS scores of 3 to 4.
5. For SAS scores persistently > 5, additional adjuvant therapies including Olanzepine®, Haldol™, Respirodone® are permitted at the discretion of the attending physician.

**Undersedation (SAS scores of 5, 6 and 7) with continuous IV sedation/analgesia**

1. With SAS scores of 5 to 7, a bolus of short acting agent (sedative/analgesic) should be administered and consideration should be given to increasing the infusion (sedative/analgesic) by approximately 50% (i.e. from Midazolam 2 mg/hr to 3 mg/hr) to achieve a SAS score of 3 to 4 at the discretion of the attending physician.
2. If the SAS score remains persistently > 5 despite at least 2 boluses and at least 2 attempts to increase infusion rates, consideration should be given to adding adjuvant antipsychotic therapy.
3. For SAS scores persistently > 5, additional adjuvant therapies including Olanzepine®, Haldol™, Respirodone® are permitted at the discretion of the attending physician.

**Appendix 4: PEEP/FiO_2_ Titration Chart (Both Study Groups)**

This scale is followed with the objective of obtaining PaO_2_ between 60 and 80 mm Hg or SpO_2_ between 90 and 95% (or at baseline saturation in chronically hypoxemic and hypercarbic patients) in both groups. SpO_2_ is assessed at least every 4 - 6 hours and arterial blood gases are performed at the discretion of the RRT/physician at least once per day. The PEEP/FiO_2_ chart is not mandatory if the patient is on an alternate mode of MV.

**Acceptable FiO_2_ and PEEP pairs are:**

| **FiO_2_** | **PEEP (in H_2_O)** |
| --- | --- |
| **0.30** | **5 cm H_2_O** |
| **0.40** | **5 cm H_2_O** |
| **0.50** | **5, 6, 7, or 8 cm H_2_O** |
| **0.60** | **8, 9 or 10 cm H_2_O** |

**Appendix 5: Other Important Considerations**

**A) Use of Noninvasive Ventilation After Extubation:**

In the event that respiratory distress develops after extubation, patients may be treated with NIV if the criteria for initiation are achieved.

**Criteria for Initiation of Noninvasive Positive Pressure Ventilation**

Initiation Criteria: The presence of any two of the following criteria are used to initiate NIV [36,37]

(i) clinical signs of respiratory distress/muscle fatigue and RR > 30 breaths/min or a 50% increase in RR from baseline,

(ii) respiratory acidosis (pH < 7.35 with PaCO_2_ > 45 mmHg),

(iii) hypoxemia (SpO_2_ < 90% or PaO_2_ < 60mmHg with FiO_2_ > 50%)

For patients randomized to AW, the AW system is not be used to manage NIV.

Initiation Technique: NIV is delivered with the patient in a seated position and the upper body elevated at a minimum of 30°. The patient-ventilator interface is fitted to the patient with oronasal masks preferred over nasal masks. Patients are encouraged to hold the mask up to their face before securing it with head straps. Excessive tension on the straps is avoided. A nasal hydrocolloid protection dressing is used to minimize discomfort related to mask application. Initial breaths are made with inspiratory pressure of 8 cm H_2_O and no expiratory pressure. In the first 2 - 5 minutes, progressive increases in inspiratory and expiratory pressure are made in increments of 2 cm H_2_O. PEEP (or expiratory positive airway pressure) is increased to a maximum of 3 cm H_2_O in COPD patients and 10 cm H_2_O in hypoxemic patients and for treatment of atelectasis. Heated humidification is preferentially used in patients, especially those with persistent hypercapnia. A variety of interfaces (different shapes/sizes) are available to minimize leaks and ensure patient comfort. Leaks and asynchronies are minimized.

Regardless of treatment assignment, the total duration of mechanical ventilation includes the time spent on NIV.

**B) Reintubation and Reinitation of Invasive Weaning:**

We use objective criteria to guide reintubation during the pilot RCT. Reintubated patients are ventilated according to initial treatment assignment once the criteria for a PST are re-attained until successful extubation, ICU death, ICU discharge or until day 90 after randomization.

**Reintubation Criteria**

Criteria for Reintubation/Reconnection in Tracheostomized Patients:

Intubation is indicated if at least one major criterion or two minor criteria are met [38]:

**Major criteria:** (i) cardiac arrest, (ii) respiratory pauses with loss of consciousness or gasping for air, (iii) psychomotor agitation interfering with nursing care, (iv) heart rate < 50 bpm with loss of alterness or (v) hemodynamic instability with systolic pressure < 80 mmHg for > 30 minutes despite adequate volume challenge, use of vasopressors or both.

For patients on NIV: (vi) a change in mental status (decrease in LOC, severe agitation) rendering the patient unable to tolerate NIV, (vii) persistent or worsening signs of respiratory distress/muscle fatigue despite NIV, (viii) abundant secretions that can not be effectively cleared or are associated with acidosis, hypoxemia or a change in mental status with NIV, (ix) failure to improve pH or PaCO_2_ with NIV.

**Minor criteria:** (i) RR >35 breaths/min and exceeding RR after extubation, (ii) pH < 7.30 and less than the value after extubation, (iii) sustained decrease in SpO_2_ < 85% despite high FiO_2_ (> 0.80) or (iv) decreasing level of consciousness (deterioration from baseline).

**C) Tracheostomy:**

Tracheostomy may be performed in either group when clinically indicated. Notwithstanding, we encourage clinicians to delay decisions regarding elective tracheostomy until day 10 after randomization.
